# Supplementary material for: Modelling global trade with optimal transport
Source: Nat Commun. 2026 Feb 19;17:2947. doi: 10.1038/s41467-026-69694-5 (PMC13031949; doi:10.1038/s41467-026-69694-5)
Supplement: Supplementary file 1 — Supplementary Information [file 41467_2026_69694_MOESM1_ESM.pdf]

Supporting Information

Validation of the approach on synthetic data

We first validate the neural inference approach on a synthetic, noiseless, gapless transport plan $\mathbf{T}$ with marginals $\boldsymbol{\mu}$ and $\boldsymbol{\nu}$, generated from the forward OT model with entropy regularisation $\varepsilon = 0.15$, and infer the cost matrix. The diagonal of the transport plan is set to 0, and the cost constrained to $[0, 1]$ by using a sigmoid activation function on the output layer of the neural network. By requiring the neural network to map the diagonal of $\mathbf{T}$ to the maximum permissible cost of 1, the cost is uniquely inferred from $\mathbf{T}$ (Fig. S1). Training is performed using the loss function described in the main manuscript, eq. (19).

To analyse robustness with regard to missing values of $\mathbf{T}$, we mask a random number of entries in the transport plan, and re-infer the cost matrix $\mathbf{C}$, given the marginals of the unmasked transport plan. Results are shown in Fig. S2. The method is robust for small amounts of missing data, since the number of gaps in each row and column are small, thus constraining how the missing "mass" contained in the marginals can be distributed among the missing entries. The error on the non-masked values of $\mathbf{T}$ remains approximately constant (which is unsurprising), while the error on the entire cost matrix increases linearly with the masking fraction, though the error on the unmasked edges remains considerably smaller (red). For the FAO data, we estimate the number of missing data points from the number of entries that have exporter- but not importer-reported figures (or vice versa); on average, this gives a missing data fraction of around 20% (see Fig. S2).

Lastly, we analyse the effect of the choice of the entropy regulariser ε on inference performance. ε is a scaling parameter that determines how much small costs affect the transport plan (Fig. S3), and can take any value in $[0, 1]$. One would like ε to be as small as possible, since for $\varepsilon \to 0$ the inference procedure converges to classical OT. However, for small values of ε Sinkhorn's algorithm becomes unstable, because as the entries of the initial guess $\exp(-\mathbf{C}/\varepsilon)$ go to 0, the required scaling vectors need to grow exponentially to match the marginal constraints. This also causes the convergence rate of Sinkhorn's algorithm to slow rapidly as $\varepsilon \to 0$, increasing the computational cost. We therefore choose a small value of $\varepsilon \approx 0.1$ that balances numerical stability and computational cost. The inference accuracy is independent of the choice of ε, see Fig. S3.

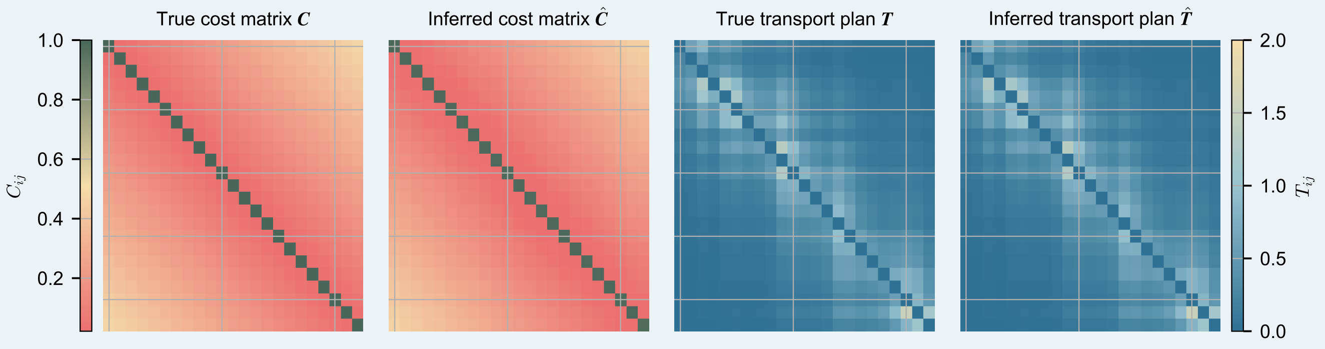

Figure S1: Inferring the cost matrix on noiseless, gapless, synthetic data. When requiring $C_{ij} \in [0, 1]$ and $u(0) = 1 = C_{max}$, the cost matrix $\mathbf{C}$ can be uniquely inferred from observations of the transport plan.

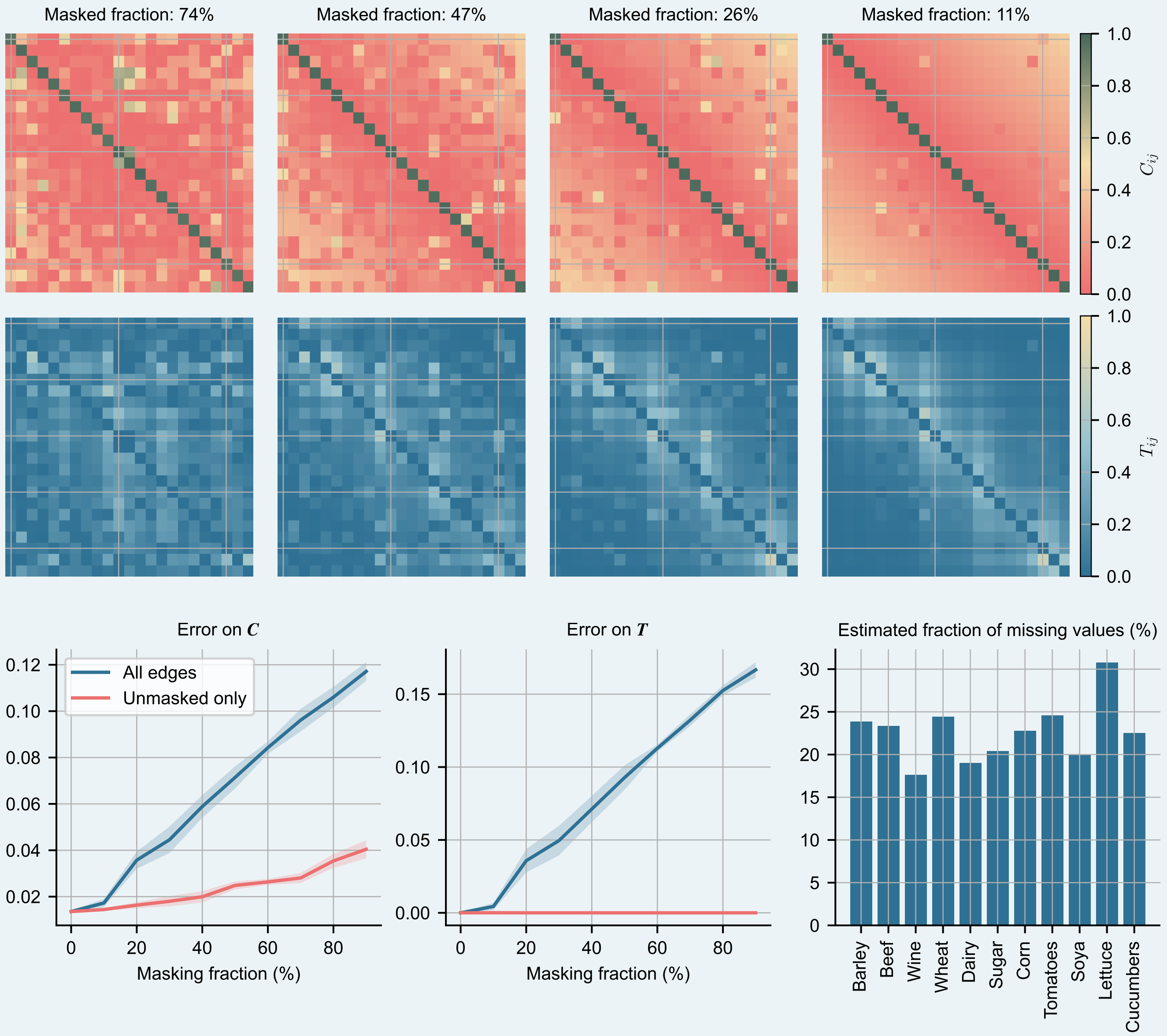

Figure S2: Top row: we infer the cost matrix on noiseless synthetic data with different fractions of the transport plan masked. Middle row: inferred transport plans. The ground truth is the same as in figure S1. Bottom row: As the fraction of masked values increases, the average L^1 error on $\mathbf{T}$ and $\mathbf{C}$ increases (blue). However, on the non-masked values, errors remain markedly lower, and the prediction on the training values of $\mathbf{T}$ is independent of the proportion of masked values (red). Shown are mean and median values, as well as the standard deviation, over all entries of the respective matrices. Right: the estimated fraction of missing values in each FAO dataset. This is estimated by comparing the number of entries reported by one reporter (exporter/importer) but not the other.

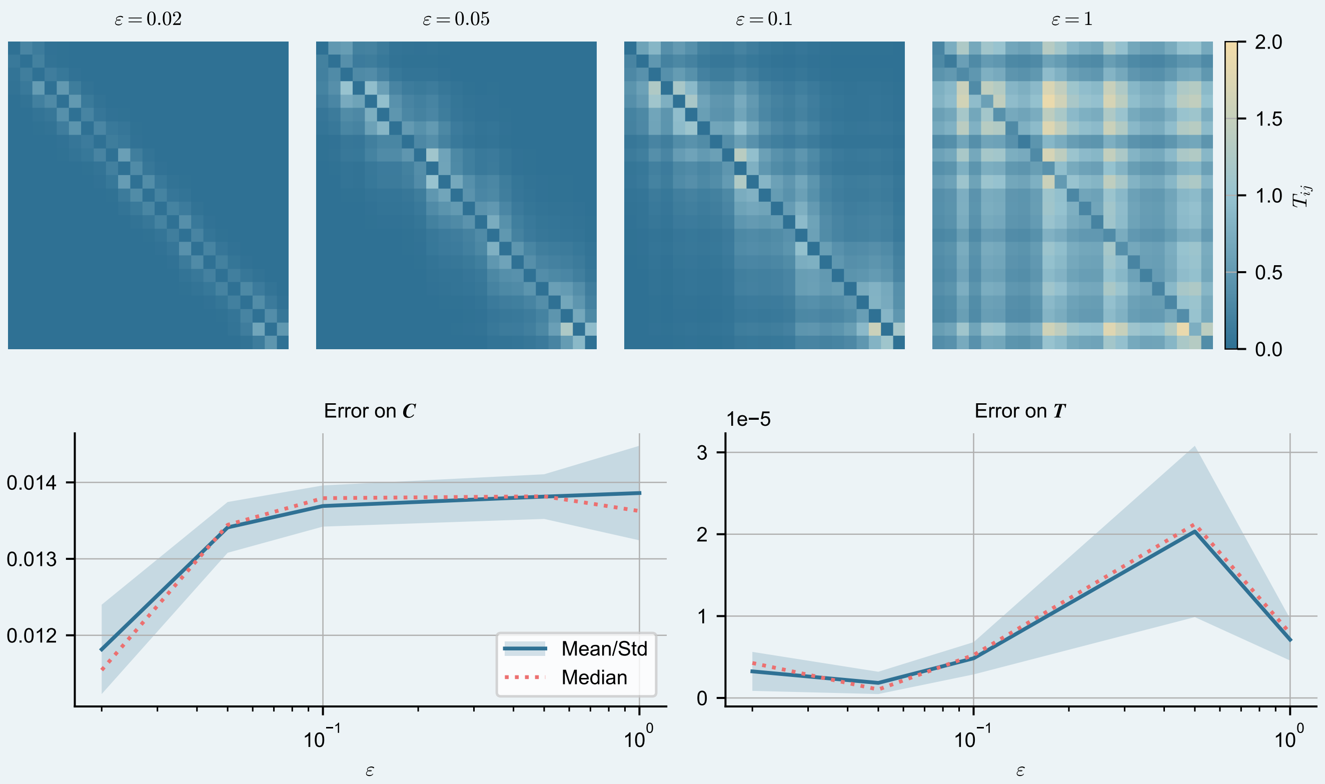

Figure S3: Effect of the entropy regulariser ε on the inference. Top row: the transport plan for the cost matrix given in fig. S1 but diferent values of ε. Bottom row: accuracy on the inferred transport plan and cost matrix as a function of the regulariser ε. Each line is an average over 5 different seeds.

Uncertainty quantification

Fig. S4 illustrates our approach to uncertainty quantification. Shown is the US-China soya bean trade (Fig. 4**C** in the main manuscript). The training data consists of exporter-reported values (top row) and importer-reported values (middle row). A family of neural networks produces a distribution of cost matrices that optimally reproduce the trade data (right column). The final distribution is generated by randomly sampling exporter- and importer-reported data points, passing them through the neural network ensemble, and averaging over the resulting cost matrices. The distribution over the cost matrices (bottom row, right) captures the uncertainty on the trade data (bottom row, left).

Case study I: the impact of the war in Ukraine on wheat trade

Here we show additional plots pertinent to our analysis of the global wheat market's response to the war in Ukraine. Figure S6 is the equivalent of figure 1 in the main manuscript, but with Russia as the exporting partner. We see a similar trend of the increase in cost being disproportionately borne by low- and lower-income countries. We substantiate this claim more thoroughly in figs. S5, S7, and S8, which show changes in trade costs and trade volumes for each geographic region, as well as as a function of per-capita GDP. Figure S5 shows the change in trade costs (left) and relative change in trade volumes (right) from both Ukraine and Russia as a function of per-capita GDP, colour-coded by region. We see that European countries appear overrepresented among the countries that saw the largest drops in cost, while Sub-Saharan Africa is overrepresented among those with the largest increases in cost. The Spearman coefficient between change in cost and GDP/capita is -0.23,

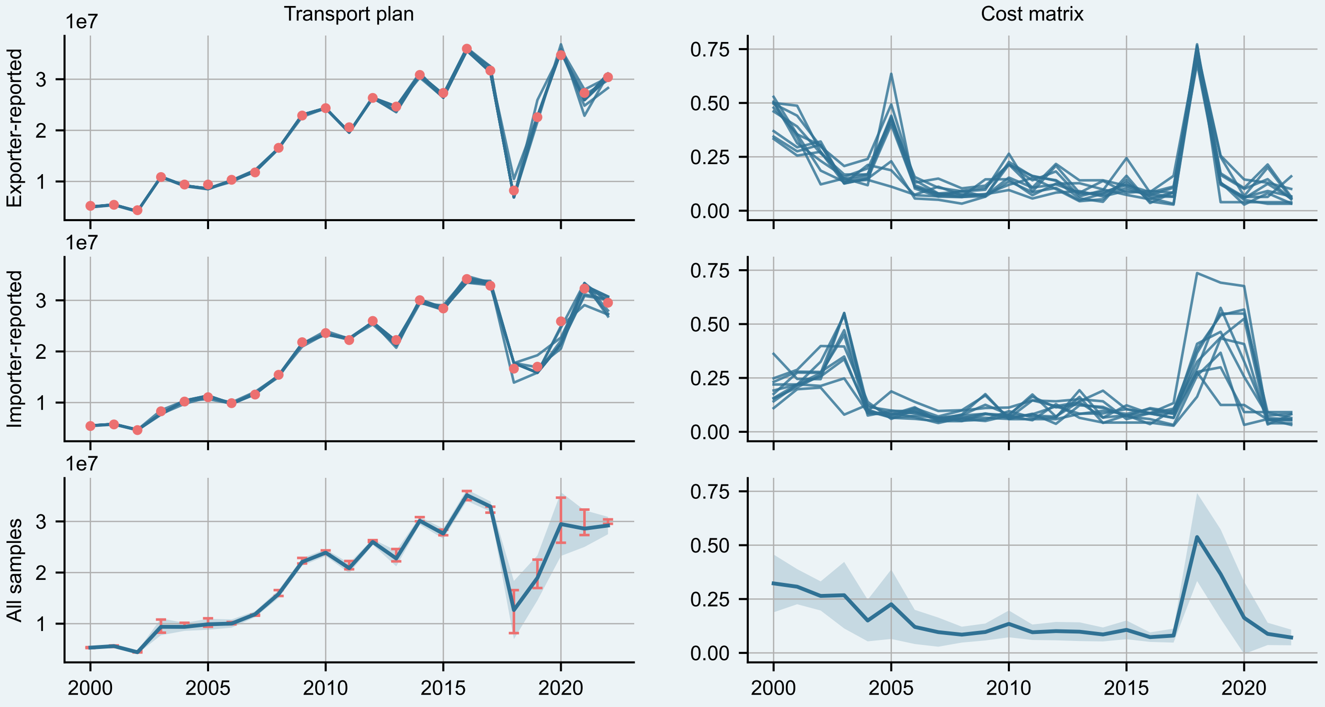

Figure S4: Constructing the distribution on the cost matrix. A family of neural networks is trained on exporter-reported values (top) and importer-reported values (middle row), producing a distribution on the cost matrix due to the potential non-uniqueness of the minimizer. Bottom row: transport plan samples are generated by randomly mixing exporter- and importer-reported values and passing these through the neural network ensemble to produce the final distribution on $\mathbf{C}$ (right).

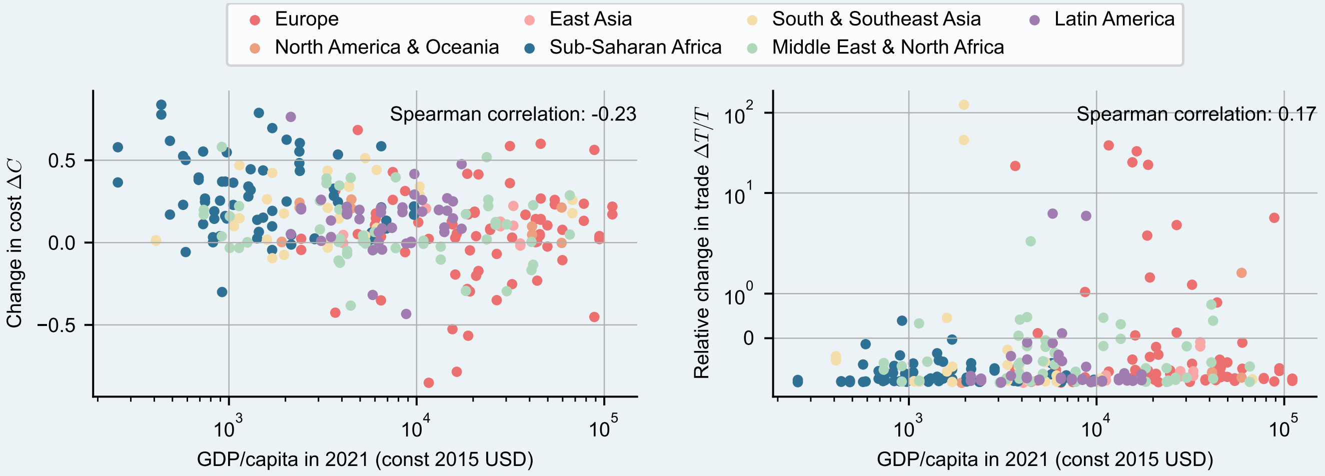

Figure S5: Changes in trade costs (left) and relative change in trade volumes (right) with both Ukraine and Russia as a function of GDP per capita. Each dot represents the change for a single importing country, colour-coded by geographic region. Also shown are the Spearman correlation coefficients.

again indicating that high and upper-middle income countries saw smaller increases in trade costs as a consequence of the war. This tallies with the fact that relative change in imports correlates positively with GDP/capita (right).

Figure S7 shows the relative change in trade volumes $\Delta T/T$ from 2021–2022 and the associated drop in trade costs for each country, disaggregated by region. Europe, for instance, experienced a median 68% decrease in trade volume and a 4%

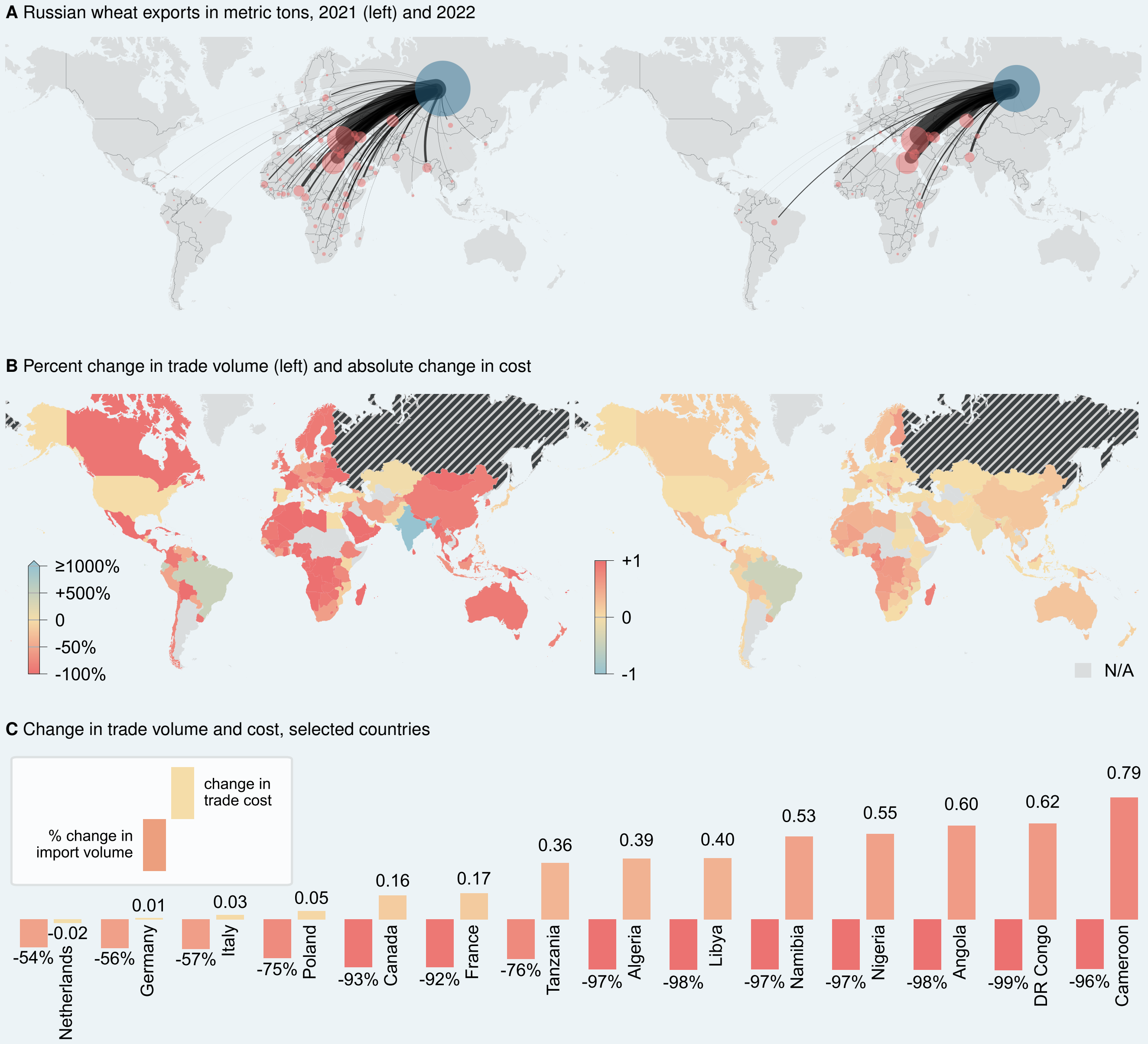

Figure S6: Russian wheat exports, 2021–2022; this plot is the equivalent to figure 1 in the main manuscript. **A** Network of Russian exports, 2021 and 2022. Shown are the largest trading partners, making up 99% of Russian exports. The blue node represents the total Russian export volume (in metric tons), the red nodes are the import volumes. Edge widths represent the flow volume. **B** The change in trade volume (left) and trade cost (right) for the largest trading partners. The large relative increase in trade to India is small in absolute terms and statistically not significant. Countries in grey either have no trade data for the period shown, or are below the 99% pooling limit (see Methods). **C** Percent change in trade volume (left bar) and change in trade cost (right bar) for selected countries.

increase in trade costs from Ukraine; North America saw a 91% decrease in trade volume and a 5% increase in trade costs. This contrasts with Sub-Saharan Africa (91% decrease in trade, 21% increase in costs), Latin America (92% decrease in trade, 14% increase in costs), or South and Southeast Asia (86% decrease in trade, 22% increase in costs). A similar, though slightly less pronounced pattern holds for Russian imports (bottom rows of each panel): here again, Sub-Saharan Africa saw the largest increase in costs, despite seeing a drop in trade comparable to that of Northern America and Oceania.

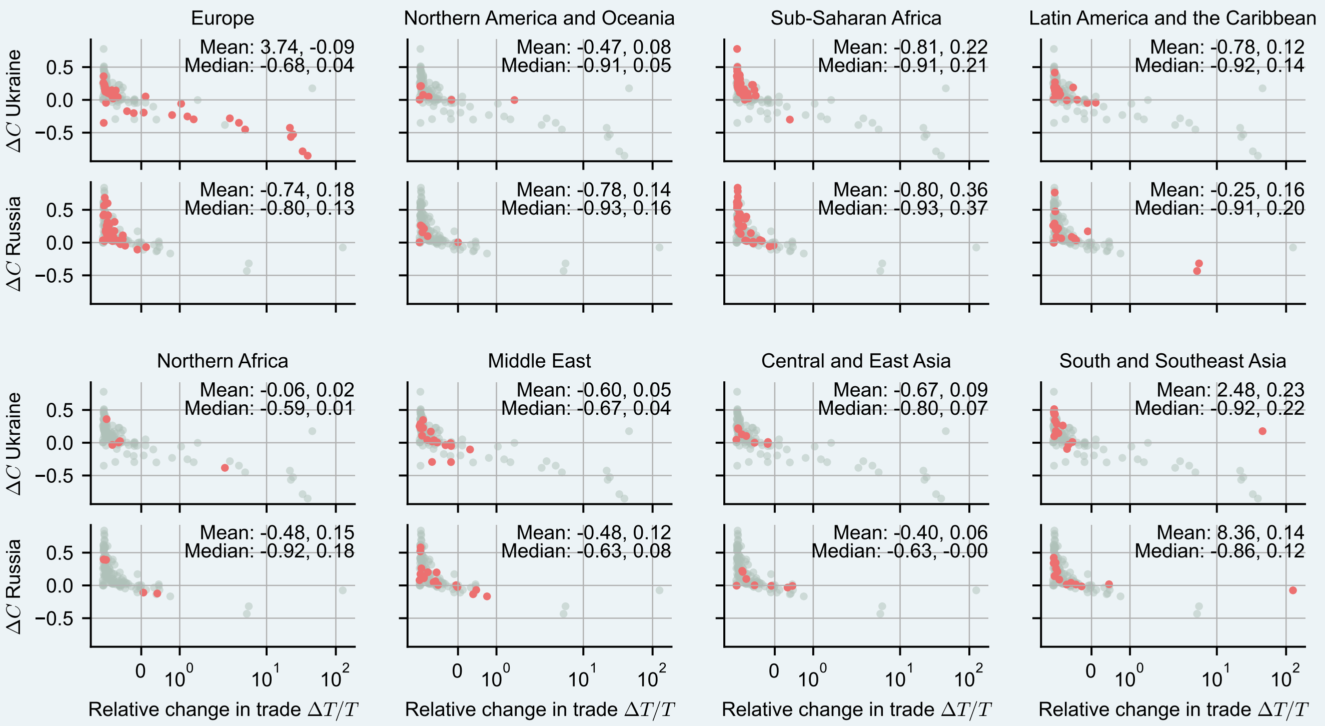

Figure S7: Change in trade cost (y-axis) as a function of the relative change in trade volume (x-axis) for each region. The top rows show the change for Ukraine as the exporter, the bottom rows show the change for Russia as the exporter. Also given are the mean and median values for $\Delta T/T$ and ΔC, respectively.

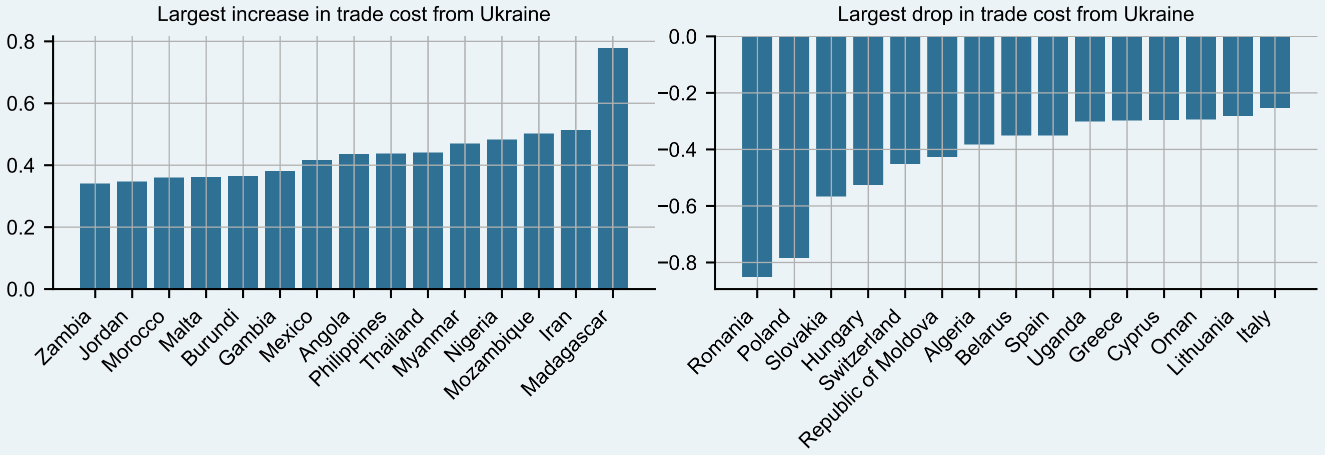

Figure S8: The top 15 countries that experienced the largest increases (left) and largest drops (right) in trade costs with Ukraine, 2021–2022.

Figure S8 shows the 15 countries that experienced the largest drops and largest increases in trade costs with Ukraine between 2021–2022.

	λ_1	λ_2	λ_3	λ_4	λ_5	λ_6	λ_7	λ_8	λ_9
Barley	0.61 (0.071)	0.085 (0.017)	-0.43 (0.017)	1.2 (0.093)	-0.44 (0.12)	0.72 (0.084)	1.3 (0.1)	0.66 (0.056)	-0.0058 (0.023)
Beef	0.34 (0.041)	0.18 (0.015)	-0.19 (0.017)	1.7 (0.057)	-0.29 (0.068)	0.78 (0.079)	1.5 (0.068)	0.55 (0.035)	0.046 (0.017)
Corn	0.86 (0.042)	0.11 (0.012)	-0.49 (0.018)	1.6 (0.077)	-0.67 (0.13)	0.44 (0.081)	1.4 (0.064)	0.72 (0.059)	-0.0058 (0.023)
Cucumbers	0.24 (0.047)	0.27 (0.024)	-0.57 (0.023)	1.8 (0.086)	-0.68 (0.1)	1.6 (0.082)	3.4 (0.21)	0.46 (0.047)	0.003 (0.012)
Dairy products*	0.58 (0.064)	0.15 (0.0089)	-0.19 (0.0099)	1.5 (0.055)	-0.11 (0.056)	0.79 (0.042)	1.9 (0.044)	0.38 (0.026)	-0.066 (0.015)
Lettuce	0.37 (0.044)	0.13 (0.015)	-0.34 (0.014)	1.9 (0.064)	-0.3 (0.085)	0.7 (0.059)	2.7 (0.12)	0.31 (0.039)	-0.029 (0.014)
Soya	0.81 (0.073)	0.21 (0.019)	-0.069 (0.023)	1.8 (0.13)	-0.84 (0.13)	1.1 (0.16)	1.2 (0.091)	0.71 (0.039)	-0.053 (0.034)
Sugar products†	0.33 (0.033)	0.094 (0.0077)	-0.33 (0.0086)	2 (0.046)	0.25 (0.051)	0.46 (0.043)	1.9 (0.038)	0.58 (0.029)	0.014 (0.012)
Tomatoes	0.28 (0.063)	0.15 (0.017)	-0.5 (0.021)	1.8 (0.081)	-0.69 (0.11)	2.2 (0.2)	3 (0.14)	0.31 (0.032)	0.019 (0.018)
Wheat	0.74 (0.042)	0.091 (0.0073)	-0.41 (0.011)	1.6 (0.055)	0.59 (0.071)	0.63 (0.048)	1.2 (0.035)	0.56 (0.029)	-0.012 (0.013)
Wine	0.39 (0.077)	0.18 (0.013)	-0.053 (0.012)	1 (0.06)	0.33 (0.06)	1.1 (0.046)	0.88 (0.048)	0.34 (0.027)	-0.024 (0.019)

Table S1: Estimated coefficients for each of the covariates used in the gravity model (1), for each commodity. Standard errors are given in parentheses. The high-dimensional coefficients κ_i, ω_j, and α_t are not shown.

*Dairy products comprise: butter, skim milk of cows, cheese, other dairy products. †Sugar products comprise: sugar, refined sugar, syrups, fructose, sugar confectionery.

Comparison with Gravity model

We consider the following gravity model specification in the spirit of traditional gravity estimations, where the covariates are based on [1]:

$$T_{i,j,t,l} = \exp(\kappa_{i,l} + \omega_{j,l} + \alpha_{t,l} + \lambda_1 \log O_{i,t,l} + \lambda_2 \log E_{j,t,l} + \lambda_3 \log d_{i,j} + \lambda_4 \mathrm{CNTG}_{i,j} + \lambda_5 \mathrm{CNLY}_{i,j} + \lambda_6 \mathrm{LANG}_{i,j} + \lambda_7 \mathrm{RTA}_{i,j} + \lambda_8 \log \chi_{j,t,l} + \lambda_9 \log \mathrm{TRFF}_{i,j,t,l})\varepsilon_{i,j,t,l}. \tag{1}$$

The covariates 5–9 are taken from the CEPII database [2]:

1. $\kappa_{i,l}$ are the exporter-fixed effects,
2. $\omega_{i,l}$ are the importer-fixed effects,
3. $\alpha_{t,l}$ are the year-fixed effects,
4. $O_{i,t,l}$ is the total production output, in tonnes, of the exporter i of product l at time t as given by the FAO,
5. $E_{j,t,l}$ is the total consumption of the importer j of product l at time t, in tonnes,
6. $d_{i,j}$ is the geodesic distance in km between the population centres (harmonic average) of countries i and j (`distw_harmonic`),

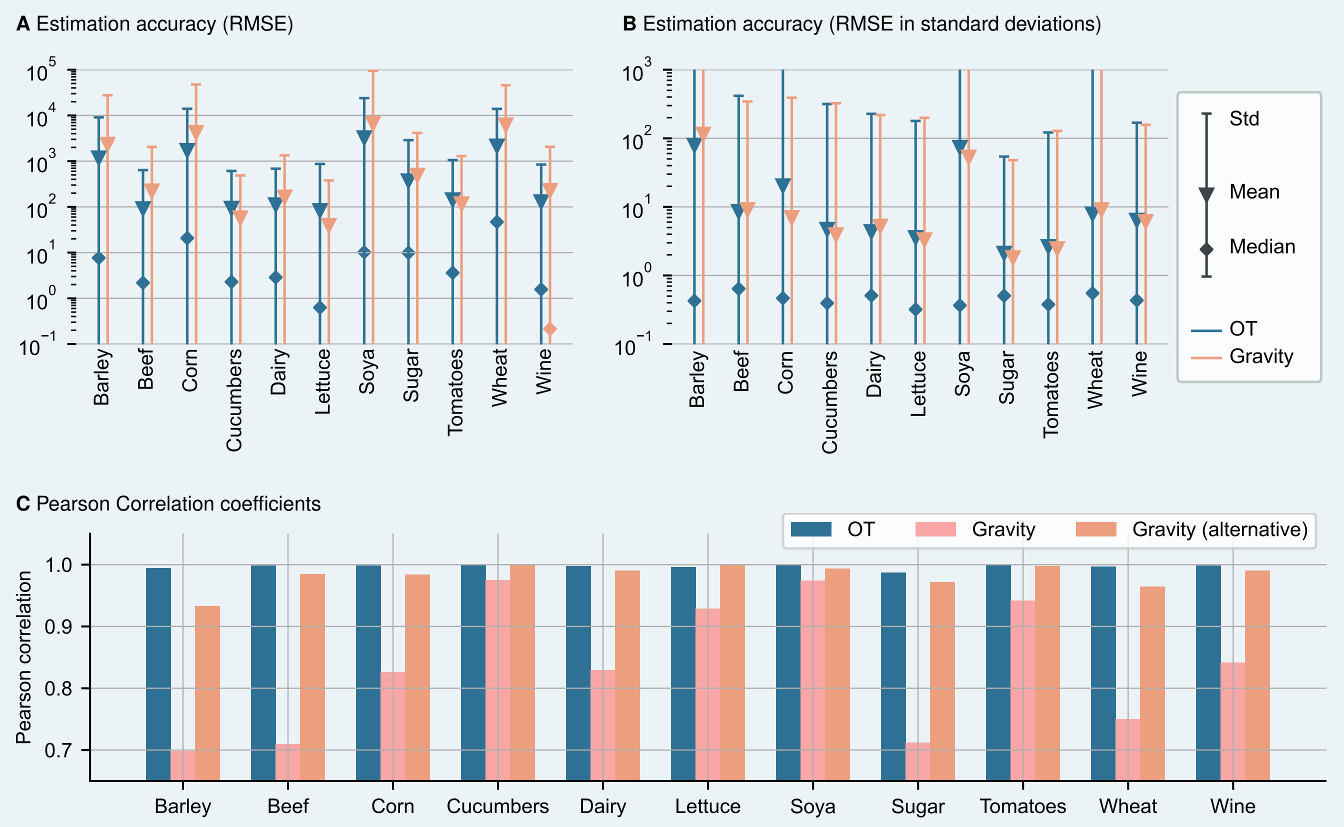

Figure S9: A–B The same plots as in Fig. 6C–D in the main manuscript, using the alternative three-way gravity model (2). The y-axis scaling is preserved. Based on the median RMSE, the three-way gravity model outperforms OT, whereas OT performs better in terms of the mean. This reflects the greater weighting—and thus lower error—for large flows, as shown in Figure S10. **C** Pearson correlation coefficients between observed and estimated transport flows for OT and both gravity models (eqs. (1) and (2)).

7. $\mathrm{CNTG}_{i,j}$ is the binary variable that indicates whether countries i and j share a land border (`contig`),

8. $\mathrm{CNLY}_{i,j}$ is a binary variable indicating whether there ever existed colonial ties before 1948 between the two trading partners (`col_dep_ever`),

9. $\mathrm{LANG}_{i,j}$ indicates whether countries i and j share an official or primary language (`comlang_off`),

10. $\mathrm{RTA}_{i,j,t}$ is a binary variable indicating whether there exists a regional trade agreement between countries i and j at time t (`rta_coverage`),

11. $\chi_{j,t,l} = \sum_i d_{i,j} O_{i,t,l} / \sum_k O_{k,t,l}$ is the remoteness index of the importer,

12. $\mathrm{TRFF}_{j,t,l}$ is the tariff applied by the importer j to product l at time t in the absence of a trade agreement. We use the most favoured nation tariff (maximum duty) as given by the WTO [3]: `MFN - Maximum duty by product groups`.

The remoteness index and the exporter/importer fixed effects account for the multilateral resistance terms [1]. This gives a $L + m + n + 9$-dimensional regression problem for each commodity, where $L = 22, m, n$ denote the number of years, exporter countries, and importer countries in the dataset (note that the regressors only span the period until 2021).

We estimate the parameters of the gravity equations using the Poisson Pseudo Maximum Likelihood (PPML) estimator [1, 4]. Data of intra-national flows and zero trade flows are excluded for consistency with the optimal transport framework. We use the PPML with high-dimensional fixed effects implementation developed in [5]. Table S1 gives the estimated parameters for each commodity. Figure S10 plots the estimated values $\hat{T}_{ij}$ against the reporter-averaged FAOStat values for both the OT and the gravity models. Also shown are a linear fit with slopes and Pearson coefficients indicated.

As an alternative specification, we use the three-way gravity model with exporter-time, importer-time, and pair (exporter-importer) fixed effects, absorbing exporter or importer level covariates, except for bilateral time variant trade policy variables, into the fixed effects, leading to:

$$T_{i,j,t,l} = \exp(\gamma_{i,t,l} + \sigma_{j,t,l} + \beta_{i,j,l} + \lambda \mathrm{RTA}_{i,j,t})\varepsilon_{i,j,t,l}. \quad (2)$$

This specification has the highest level of flexibility, using regressors only for time-dependent bilateral trade terms (participation in regional trade agreements). The country-time fixed effects capture both the time-dependent pull effect due to the size and the multilateral resistance terms, while the pair effect captures time-invariant bilateral effects [6]. This alternative specification has $L \times (m+n) + m \times n + 1$ parameters (see Fig. S9).

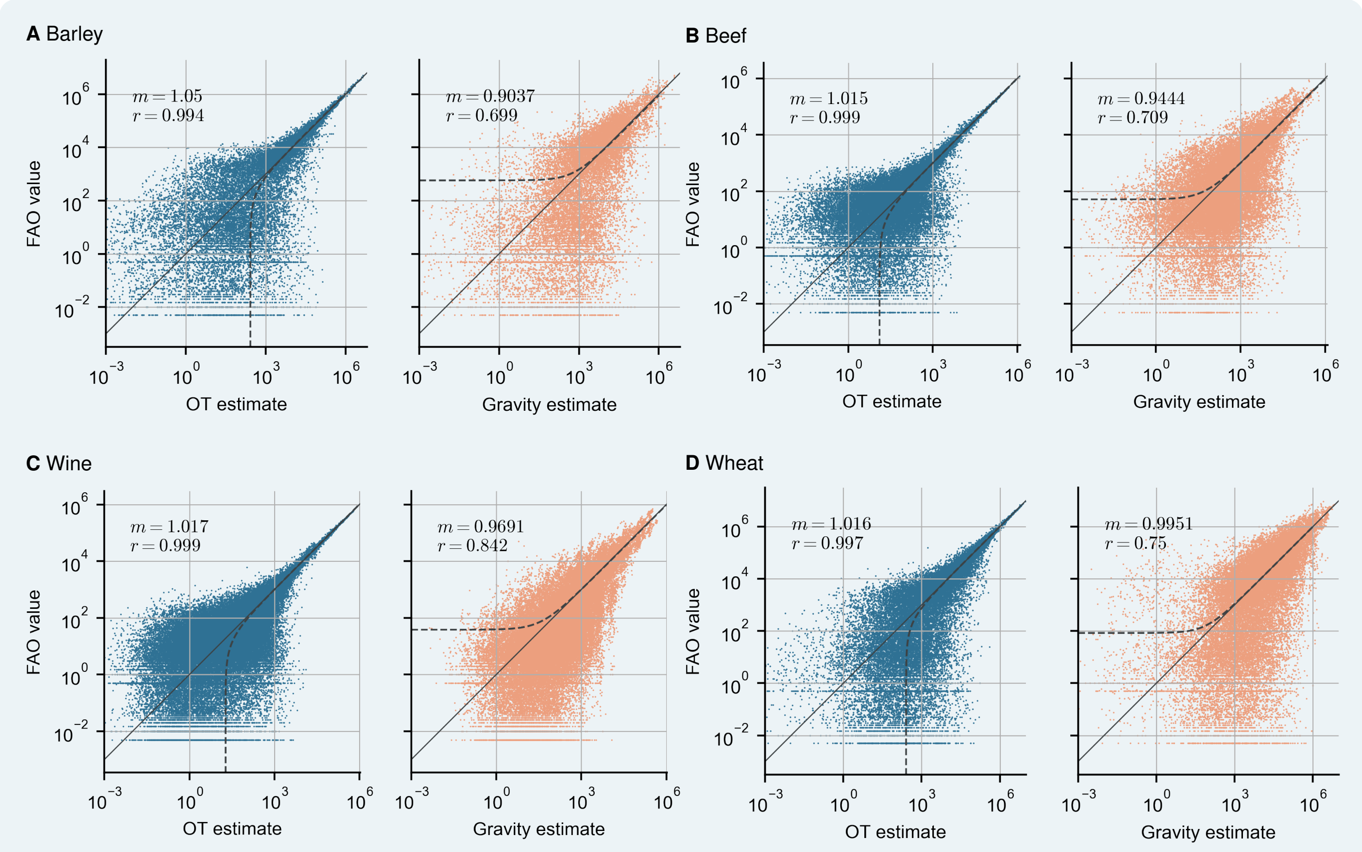

(continued on next page)

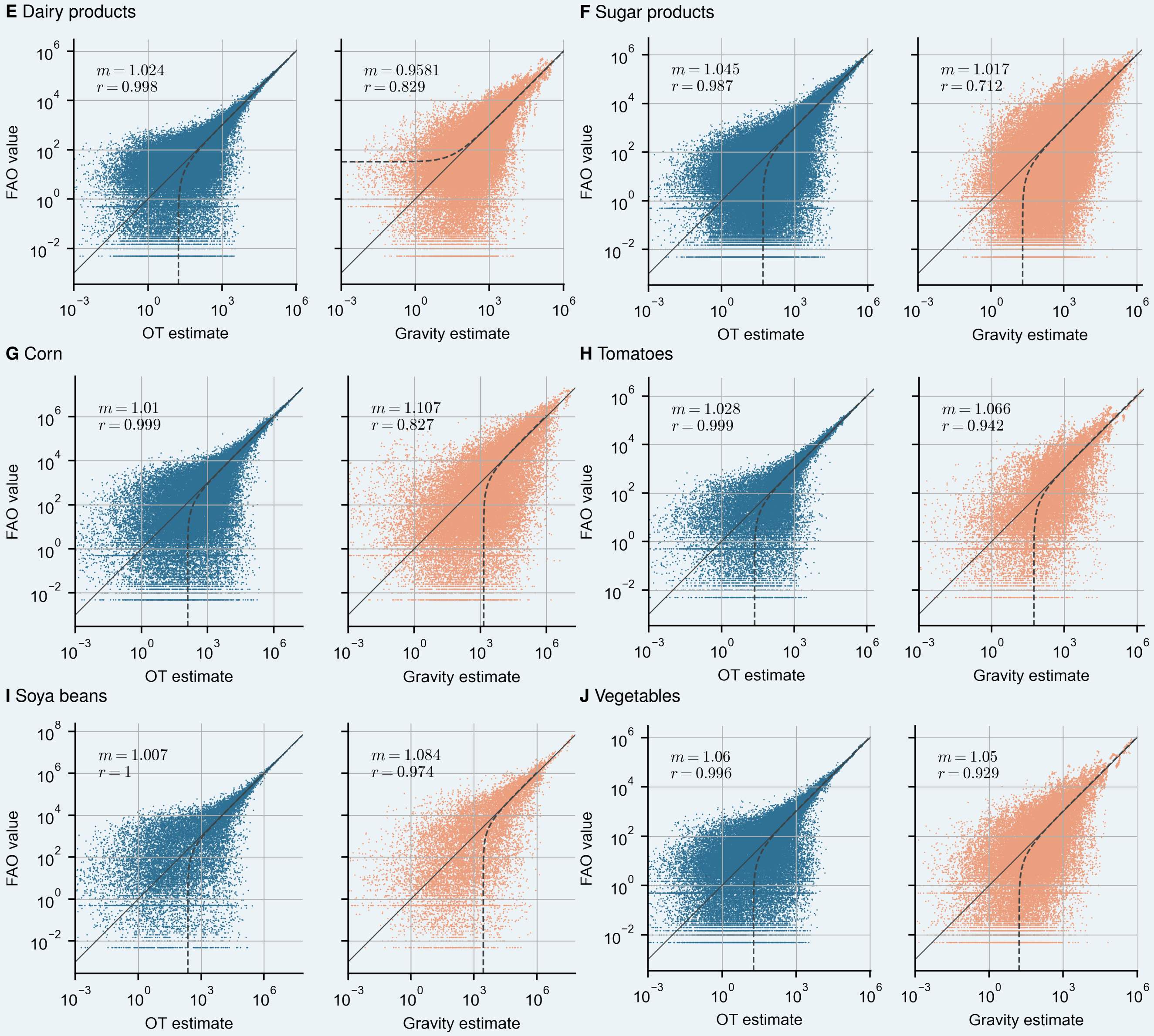

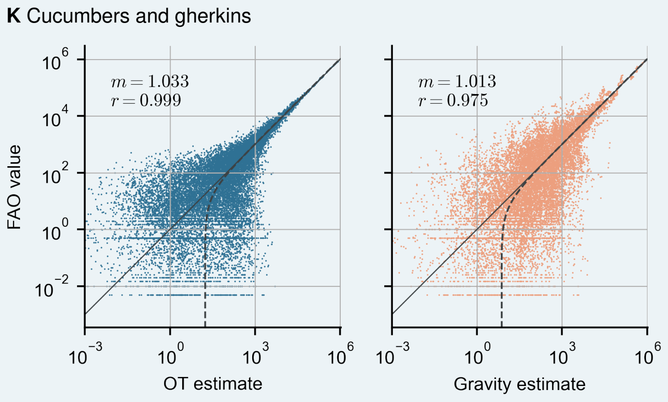

Figure S10: Comparison of the OT estimates (left, darkblue) and the Gravity estimates (eq. (1), for each commodity. The y-axis shows the true FAO value, while the x-axis shows the estimated value. The solid line is the diagonal $y = x$. Also shown are a linear fit (dashed line) as well the fitted slope m and Pearson correlation r of the fit. **A** Barley. **B** Beef. **C** Wine. **D** Wheat. **E** Dairy products, comprising butter, skim milk of cows, cheese, other dairy products. **F** Sugar products, comprising sugar, refined sugar, syrups, fructose, sugar confectionery. **G** Corn. **H** Tomatoes. **I** Soya beans. **J** Vegetables. **K** Cucumbers and gherkins.
