# Supplementary material for: Modelling global trade with optimal transport
Source: Nat Commun. 2026 Feb 19;17:2947. doi: 10.1038/s41467-026-69694-5 (PMC13031949; doi:10.1038/s41467-026-69694-5)
Supplement: Supplementary file 2 — Reporting Summary [file 41467_2026_69694_MOESM2_ESM.pdf]

nature portfolio

Corresponding author(s): Thomas Gaskin

Last updated by author(s): Dec 12, 2025

Reporting Summary

Nature Portfolio wishes to improve the reproducibility of the work that we publish. This form provides structure for consistency and transparency in reporting. For further information on Nature Portfolio policies, see our Editorial Policies and the Editorial Policy Checklist.

Statistics

For all statistical analyses, confirm that the following items are present in the figure legend, table legend, main text, or Methods section.

n/a	Confirmed	
☐	☒	The exact sample size (n) for each experimental group/condition, given as a discrete number and unit of measurement
☐	☒	A statement on whether measurements were taken from distinct samples or whether the same sample was measured repeatedly
☒	☐	The statistical test(s) used AND whether they are one- or two-sided<br>*Only common tests should be described solely by name; describe more complex techniques in the Methods section.*
☐	☒	A description of all covariates tested
☒	☐	A description of any assumptions or corrections, such as tests of normality and adjustment for multiple comparisons
☐	☒	A full description of the statistical parameters including central tendency (e.g. means) or other basic estimates (e.g. regression coefficient) AND variation (e.g. standard deviation) or associated estimates of uncertainty (e.g. confidence intervals)
☒	☐	For null hypothesis testing, the test statistic (e.g. F, t, r) with confidence intervals, effect sizes, degrees of freedom and P value noted<br>*Give P values as exact values whenever suitable.*
☒	☐	For Bayesian analysis, information on the choice of priors and Markov chain Monte Carlo settings
☒	☐	For hierarchical and complex designs, identification of the appropriate level for tests and full reporting of outcomes
☐	☒	Estimates of effect sizes (e.g. Cohen's d, Pearson's r), indicating how they were calculated

Our web collection on statistics for biologists contains articles on many of the points above.

Software and code

Policy information about availability of computer code

Data collection	FAOStat: detailed trade matrix, as of 2022, yield and total production figures: https://www.fao.org/faostat/en/#data<br>GDP data: World Bank data (indicator NY.GDP.MKTP.KD): https://data.worldbank.org/indicator/NY.GDP.MKTP.KD
Data analysis	PyTorch ≥ 2.8; Python ≥3.9; Standard Python packages; all code and requirements available at Github.

For manuscripts utilizing custom algorithms or software that are central to the research but not yet described in published literature, software must be made available to editors and reviewers. We strongly encourage code deposition in a community repository (e.g. GitHub). See the Nature Portfolio guidelines for submitting code & software for further information.

Data

Policy information about availability of data

All manuscripts must include a data availability statement. This statement should provide the following information, where applicable:

- Accession codes, unique identifiers, or web links for publicly available datasets
- A description of any restrictions on data availability
- For clinical datasets or third party data, please ensure that the statement adheres to our policy

Code is freely available at https://github.com/ThGaskin/inverse-optimal-transport with permanent release number v0.0.0. Instructions for obtaining the data and running the model are given in the README. Data is available at Huggingface: https://huggingface.co/datasets/ThGaskin/OT_Trade, with permanent DOI 10.57967/hf/7190.

Research involving human participants, their data, or biological material

Policy information about studies with human participants or human data. See also policy information about sex, gender (identity/presentation), and sexual orientation and race, ethnicity and racism.

Reporting on sex and gender	N/A
Reporting on race, ethnicity, or other socially relevant groupings	N/A
Population characteristics	N/A
Recruitment	N/A
Ethics oversight	N/A

Note that full information on the approval of the study protocol must also be provided in the manuscript.

Field-specific reporting

Please select the one below that is the best fit for your research. If you are not sure, read the appropriate sections before making your selection.

☐ Life sciences ☐ Behavioural & social sciences ☒ Ecological, evolutionary & environmental sciences

For a reference copy of the document with all sections, see nature.com/documents/nr-reporting-summary-flat.pdf

Ecological, evolutionary & environmental sciences study design

All studies must disclose on these points even when the disclosure is negative.

Study description	This study models and analyses global trade of food and agricultural products from 2000-2022 using optimal transport.
Research sample	We use a set of 11 commodities from FAOStat, selected mainly based on total consumption levels and importance to global food security: Wheat, Barley, Soya, Corn, Beef, Dairy, Sugar; additionally fresh produce (Cucumbers, Lettuce, Tomatoes), and a single luxury item (Wine).
Sampling strategy	Samples on the final estimates are obtained by using a trained neural network as a push forward measure. The uncertainty on the input data (FAOStat tables) is obtained by sampling importer and exporter-reported values uniformly at random and passing the generated tables through the network. In addition, we use ensemble training with 10 neural networks initialized uniformly at random.
Data collection	Data was downloaded from the FAOStat portal and WorldBank Open Data portal by T. Gaskin.
Timing and spatial scale	N/A
Data exclusions	No data was excluded, but all trade flows contributing to less than 99% of total trade were subsumed under an 'Other' category and not further analysed in the paper. These are flows whose absolute value is typically lower than the total variance of the data, or between countries that do not consistently trade a good, or between countries that do not formally exist for the entirety of the period considered. This is a relabeling exercise rather than data exclusion.
Reproducibility	All figures are fully reproducible using the Jupyter Evaluation notebook provided. All trained neural networks, including training data and trained weights, are fully available at the data repository.
Randomization	N/A
Blinding	N/A

Did the study involve field work? ☐ Yes ☒ No

Reporting for specific materials, systems and methods

We require information from authors about some types of materials, experimental systems and methods used in many studies. Here, indicate whether each material, system or method listed is relevant to your study. If you are not sure if a list item applies to your research, read the appropriate section before selecting a response.

Materials & experimental systems

n/a	Involved in the study
x	Antibodies
x	Eukaryotic cell lines
x	Palaeontology and archaeology
x	Animals and other organisms
x	Clinical data
x	Dual use research of concern
x	Plants

Methods

n/a	Involved in the study
x	ChIP-seq
x	Flow cytometry
x	MRI-based neuroimaging

Plants

Seed stocks	N/A
Novel plant genotypes	N/A
Authentication	N/A
